# Supplementary material for: Clinical and Echocardiographic Predictors for the Presence of Late Gadolinium Enhancement on Cardiac Magnetic Resonance Imaging in Patients with Carbon Monoxide Poisoning
Source: Diagnostics (Basel). 2023 Dec 27;14(1):60. doi: 10.3390/diagnostics14010060 (PMC10795751; doi:10.3390/diagnostics14010060)
Supplement: Supplementary file 1 [file diagnostics-14-00060-s001.zip › diagnostics-2722668-supplementary.pdf]

**Clinical and Echocardiographic Predictors for the Presence of Late Gadolinium Enhancement on Cardiac Magnetic Resonance Imaging in Patients with Carbon Monoxide Poisoning**

**Supplementary materials**

**Supplementary Table S1.** Predictors of LGE positivity using univariate logistic regression

**Supplementary Table S1.** Predictors of LGE positivity using univariate logistic regression.

| Variables | Univariate | | |
|---|---|---|---|
| | Standardized β | OR (95% CI) | p-value |
| **Sex** | | | |
| Male | | Ref. | |
| Female | -0.077 | 0.75 (0.38–1.47) | 0.405 |
| **Age** | | | |
| ≤45 | | Ref. | |
| >45 | -0.098 | 0.7 (0.35–1.38) | 0.299 |
| **Intentionality** | | | |
| No | | Ref. | |
| Yes | 0.002 | 1.01 (0.52–1.95) | 0.981 |
| **Source** | | | |
| Charcoal | | Ref. | |
| Gas and oil | 0.093 | 1.03 (0.42–2.51) | 0.948 |
| Fire | -0.133 | 0.55 (0.15–1.99) | 0.359 |
| **Maximal estimated CO exposure times (hours)** | | | |
| >5 | | Ref. | |
| ≤5 | -0.169 | 0.54 (0.28–1.05) | 0.069 |
| **Hypertension** | | | |
| No | | Ref. | |
| Yes | -0.010 | 0.96 (0.45–2.05) | 0.916 |
| **Diabetes mellitus** | | | |
| No | | Ref. | |
| Yes | -0.030 | 0.83 (0.28–2.48) | 0.743 |
| **Hyperlipidemia** | | | |
| No | | Ref. | |
| Yes | -0.109 | 0.43 (0.11–1.67) | 0.223 |
| **Time from rescue to $HBO_2$ (h)** | | | |
| ≤4 | | Ref. | |
| >4 | 0.177 | 2.418 (1.001–5.841) | 0.0497 |
| **Glasgow Coma Scale** | | | |
| >6 | | Ref. | |
| ≤6 | -0.105 | 0.62 (0.28–1.38) | 0.244 |

| | | | |
|---|---|---|---|
| **Loss of consciousness** | | | |
| No | | Ref. | |
| Yes | 0.017 | 1.12 (0.35–3.59) | 0.855 |
| **Shock** | | | |
| No | | Ref. | |
| Yes | 0.010 | 1.06 (0.4–2.83) | 0.911 |
| **Laboratory findings** | | | |
| **COHb (%)** | | | |
| ≤30 | | Ref. | |
| >30 | -0.101 | 0.69 (0.35–1.34) | 0.271 |
| **Creatinine (mg/dL)** | | | |
| >0.9 | | Ref. | |
| ≤0.9 | -0.155 | 0.57 (0.29–1.11) | 0.096 |
| **Creatine kinase (U/L)** | | | |
| ≤500 | | Ref. | |
| >500 | 0.190 | 1.99 (1.02–3.91) | 0.045 |
| **Initial troponin I (pg/mL)** | | | |
| >480 | | Ref. | |
| ≤480 | -0.196 | 0.49 (0.25–0.96) | 0.037 |
| **Peak troponin I (pg/mL)** | | | |
| >990 | | Ref. | |
| ≤990 | -0.184 | 0.514 (0.264–0.999) | 0.0496 |
| **BNP (pg/mL)** | | | |
| >95 | | Ref. | |
| ≤95 | -0.224 | 0.43 (0.20–0.90) | 0.024 |
| **Bicarbonate (mmol/L)** | | | |
| >20 | | Ref. | |
| ≤20 | 0.203 | 2.08 (1.07–4.07) | 0.032 |
| **Lactate (mg/dL)** | | | |
| ≤2 | | Ref. | |
| >2 | 0.231 | 2.56 (1.24–5.29) | 0.011 |
| **LVEF (%)** | | | |
| >63 | | Ref. | |
| ≤63 | 0.197 | 2.23 (1.08–4.6) | 0.030 |
| **LV GLS (%)** | | | |
| ≤-16 | | Ref. | |
| >-16 | 0.322 | 3.31 (1.57–7.02) | 0.002 |

| | | | |
|---|---|---|---|
| **LVEDV (mL)** | | | |
| ≤103 | | Ref. | |
| >103 | 0.114 | 1.52 (0.78–2.95) | 0.222 |
| **LVESV (mL)** | | | |
| ≤43 | | Ref. | |
| >43 | 0.234 | 2.34 (1.18–4.61) | 0.014 |
| **LVEDD (mm)** | | | |
| ≤49 | | Ref. | |
| >49 | 0.124 | 1.59 (0.81–3.09) | 0.176 |
| **LVESD (mm)** | | | |
| ≤29 | | Ref. | |
| >29 | 0.207 | 3.21 (1.17–8.84) | 0.024 |
| **SWT (mm)** | | | |
| >7 | | Ref. | |
| ≤7 | 0.104 | 1.5 (0.73–3.07) | 0.274 |
| **PWT (mm)** | | | |
| ≤7 | | Ref. | |
| >7 | 0.063 | 1.28 (0.64–2.56) | 0.489 |
| **LVMI (g/m²)** | | | |
| ≤70 | | Ref. | |
| >70 | 0.191 | 2.14 (1.05–4.35) | 0.036 |
| **LAVI (mL/m²)** | | | |
| ≤19 | | Ref. | |
| >19 | 0.171 | 2.43 (0.97–6.05) | 0.057 |
| **Septal e′ (cm/s)** | | | |
| ≤4 | | Ref. | |
| >4 | 0.088 | 1.58 (0.63–3.92) | 0.328 |
| **E/e′ (ratio)** | | | |
| >7 | | Ref. | |
| ≤7 | 0.152 | 1.81 (0.86–3.81) | 0.119 |
| **RVSP (mmHg)** | | | |
| ≤27 | | Ref. | |
| >27 | 0.108 | 1.49 (0.75–2.97) | 0.255 |

Abbreviations: OR = odds ratio, CI = confidence interval, CO = carbon monoxide, $HBO_2$ = hyperbaric oxygen therapy, COHb = carboxyhemoglobin, BNP = β-type natriuretic peptide, LVEF = left ventricular ejection fraction, LV GLS = left ventricular global longitudinal strain, LVEDV = left ventricular end-diastolic volume, LVESV = left ventricular end-systolic volume, LVEDD = left ventricular end-diastolic dimension,

LVESD = left ventricular end-systolic dimension, SWT = septal wall thickness, PWT = posterior wall thickness, LVMI = left ventricular mass index, LAVI = left atrial volume index, Septal e′ = septal early mitral tissue velocity, RVSP = right ventricular systolic pressure, LGE = late gadolinium enhancement.
